# Editorial: Advances in Bioanalytical Methods for Probing Ligand-Target Interactions

**DOI:** 10.3389/fchem.2020.00378

**Published:** 2020-05-15

**Authors:** Quezia B. Cass, Gabriella Massolini, Carmen Lucia Cardoso, Enrica Calleri

**Affiliations:** ^1^Departamento de Química, Universidade Federal de São Carlos, São Carlos, Brazil; ^2^Department of Drug Sciences, University of Pavia, Pavia, Italy; ^3^Departamento de Química, Faculdade de Filosofia, Ciências e Letras de Ribeirão Preto, Universidade de São Paulo, Ribeirão Preto, Brazil

**Keywords:** bioaffinity chromatography, surface plasmon resonance, nuclear magnetic resonance, mass spectrometry-based approaches, ligand-fishing experiments

Protein-ligand interactions are fundamental in all life processes and the comprehension of molecular recognition has an essential role in many scientific areas. Molecular recognition processes involve protein-protein, protein-ligand, or protein-nucleic acid interactions and are mainly driven by weak bonding interactions (hydrogen bonding, electrostatics, van der Waals forces, π-π interactions). The study of drug–target (enzymes, proteins, nucleic acids) interactions is crucial for the discovery and development of new therapeutic molecules as the binding affinity of a ligand may provide important information about *in vivo* efficacy of the compound. In this context, increased attention has been given to the development of ligand-binding assays that target protein interactions using a variety of analytical methods.

Bioaffinity chromatography/electrophoresis (Slon-Usakiewicz et al., 2005; Calleri et al., 2011; Moraes et al., 2016), ligand-fishing experiments (de Moraes et al., 2014; Liu et al., 2017; Zhang et al., 2019), mass spectrometry (MS)-based approaches (Imaduwage et al., 2016), nuclear magnetic resonance (NMR) (Cala et al., 2014; Furukawa et al., 2016), surface plasmon resonance (SPR) (Nedelkov and Nelson, 2003) and other techniques such as quartz crystal microbalance (Naklua et al., 2016), equilibrium dialysis, ultrafiltration (Zhuo et al., 2016), and circular dichroism (Tramarin et al., 2019) have been reported in literature for ligand-target interaction studies.

In this special issue, three reviews and six original research papers provide methodological advances and recent application examples in bioaffinity chromatography, MS based binding assays, SPR, and NMR for ligand-target interactions studies. In the following paragraphs, each of the accepted publications are presented and briefly described.

Regarding chromatography, the topic encompasses two closely related reviews and three application articles. The review from de Moraes et al. covers screening assays in which the binding events are monitored by on-line or off-line liquid chromatography. On-line bioaffinity chromatography, with zonal or frontal elution, in a diverse range of assay systems are presented and their applications for disclosing protein-ligand interactions is discussed. Meanwhile, a diverse range of static, or off-line, assays have been reported that identify ligands in complex mixtures. In the latter cases, the full chemical characterization of the identified ligands is the main attraction of these approaches. Tools for examining the kinetics of biological reactions, such as band-broadening measurements, peak decay analysis, split-peak and peak fitting methods, and ultrafast affinity extraction are beautifully reviewed by Iftekhar et al.. An off-line assay application is reported in the work of Sahm et al. for the TBX2 transcription factor, which plays a key role in oncogenic processes. After immobilization to an *N*-terminal resin, the TBX2-DNA-binding domain recombinant protein was used to evaluate the capability of marine chromomycins A5 (CA5) and A6 (CA6) to interact with TBX2. Microscale thermophoresis was used to characterize the interaction observed through the screen modulators static assay. Based on the evidence generated by these assays, it was possible to infer that chromomycins, and particularly CA5, bind to TBX2 and its modulation may explain their cytotoxic properties.

Olsen et al. report on the production of monolithic supports for trypsin immobilization for on-line protein digestion in bottom-up proteomic studies, which can also be tailored for other applications such as on-line drug/target studies. The article by de Lima et al. deals with molecular docking and bioaffinity chromatography as a means to design rational acetylcholinesterase (AChE) inhibitors using stepholidine as a template. In this way it was possible to kinetically characterize the identified inhibitors, and show how the designed alkaloid derivatives interact with AChE.

Mass spectrometry (MS) is recognized as a powerful tool to study the non-covalent interactions between small molecules and proteins (ligand-target interactions) that is a hot topic in medicinal chemistry and life sciences. In this special issue, readers can find an interesting review by Chen et al. where the principles and recent applications of soft ionization mass spectrometry methods (ESI, DESI, and MALDI) and their hyphenated techniques, including hydrogen-deuterium exchange mass spectrometry (HDX-MS), chemical cross-linking mass spectrometry (CX-MS), and ion mobility spectrometry-mass spectrometry (IMS/MS) are described. The Authors underline that the soft ionization MS-based methods can carefully and accurately study protein/enzyme-small molecule interactions, thus providing important information-rich data useful in drug design and development.

In an interesting article Gabriel et al. combined the concepts of MS Binding Assays and affinity selection MS (ASMS). The new, powerful, efficient, and reliable library screening approach has been used for the identification of ligands addressing the GABA transporter subtype 1 (GAT1) responsible of the regulation of GABA levels in the brain. To meet this end a library composed initially of 128, later of 1,280, well-characterized GAT1 inhibitors, drug substances, and pharmacological tool compounds were analyzed. The described approach combines the power of MS Binding Assays and the strength of ASMS, while the weaknesses of both methods are avoided. The capabilities offered by the combination of competitive MS Binding Assays and ASMS can exploited in early drug discovery campaigns.

For the characterization of a binding event and for the affinity screening of libraries of compounds as potential drug candidates, different biophysical techniques can be considered. Among the most informative techniques, surface plasmon resonance (SPR) spectroscopy is a relatively new label-free technique which enables the measurement of real-time ligand-binding affinities and kinetics using small amounts of target immobilized on a sensor-chip.

One of most challenging tasks for SPR methods is the measurement of the binding kinetics and affinities of potential ligands to membrane proteins such as GPCRs. Very few applicative examples have been reported so far. In this special issue an original contribution has been described by Capelli et al.. The article reports the development of an SPR method for the measurement of binding affinities and kinetic parameters of potential ligands for GPR17-an important target for the treatment of demyelinating diseases. The receptor was immobilized from solubilized membrane extracts through a covalently bound anti-6x-His-antibody.

The method was applied to two engineered variants of GPR17, expressed in insect cells and extracted from crude membranes and used for the characterization of the binding of two high affinity ligands, the antagonist Cangrelor and the agonist Asinex 1. The experimentally calculated kinetic parameters and binding constants were in good agreement with those reported in literature.

NMR-based spectroscopic methods can be used to characterize at an atomic level a binding interaction in aqueous media. Kock et al. reported a study where the interaction between a novel ruthenium complex anti-cancer candidate and the biological target DNA is considered. Moreover, the *K*_d_ value was estimated and it was in agreement with previously reported studies increasing the potential of the technique for medicinal chemistry programs on new metallodrugs.

The published articles help readers appreciate the usefulness of different analytical tools for studying protein-drug interactions. Researchers working in pharmaceutical industries and academia can greatly benefit from the application of the proposed innovative analytical methods to improve the drug discovery and development processes.

## Author Contributions

All authors listed have made a substantial, direct and intellectual contribution to the work, and approved it for publication.

## Conflict of Interest

The authors declare that the research was conducted in the absence of any commercial or financial relationships that could be construed as a potential conflict of interest.
